# Caregiver Burden in Caring for Family Members with Cancer in the Makkah Region, Saudi Arabia: A Cross-Sectional Study

**DOI:** 10.3390/healthcare14081113

**Published:** 2026-04-21

**Authors:** Nuha Mahdi, Hashim A. Mahdi

**Affiliations:** 1 Allied Health Postgraduate Administration, Executive Administration of Academic Affairs and Training, King Abdullah Medical City, Makkah 24246, Saudi Arabia; 2 Department of Public Health, College of Health Sciences, Saudi Electronic University, Riyadh 13316, Saudi Arabia

**Keywords:** cancer, caregiving burden, family caregiver, Saudi Arabia, Zarit Burden Interview

## Abstract

**Highlights:**

**What are the main findings?**

- Over half of family caregivers of adult cancer patients in Makkah, Saudi Arabia, reported mild-to-moderate caregiver burden.
- Financial hardship and patient immobility (bedridden status) were the strongest factors significantly associated with higher caregiver burden scores.

**What are the implications of the main findings?**

- Cancer caregiver support programs should prioritize financial counselling/support and interventions addressing the demands of caring for immobile patients.
- Routine caregiver screening and culturally responsive services can guide early identification of high-burden caregivers and improve caregiver well-being.

**Abstract:**

**Background:** The present study aimed to assess the caregiving burden among family caregivers of adult patients with various cancer types and stages in the Kingdom of Saudi Arabia (KSA), and to examine the associations with caregiver and patient characteristics. **Materials and Methods:** A cross-sectional study involving 212 family caregivers of cancer patients was conducted between March and April 2024 at King Abdullah Medical City in Makkah, KSA. The Arabic version of the Zarit Burden Interview (ZBI) scale was used to assess overall and specific burdens. Associations between overall burden and sociodemographic variables were analyzed using significance tests. **Results:** Over half (55%) of participants experienced burden, with a mean ZBI score of 26.33 ± 16.86, indicating a mild to moderate level. Low levels of psychological (7.34 ± 5.41), social (2.27 ± 2.93), physical (1.96 ± 2.22), and financial (1.22 ± 1.41) burdens were found. Financial difficulties and patient immobility significantly contributed to higher burden scores. Caregivers with financial hardships scored higher (31 ± 14.8 vs. 24 ± 17.3, *p* = 0.01), and those caring for bedridden patients experienced greater burdens (38 ± 21.8 vs. 18 ± 12.5, *p* = 0.001). **Conclusions:** Although financial difficulties and patient immobility significantly contribute to caregiver burden, the overall burden in the Makkah region remains relatively moderate. Strong cultural and familial support systems in KSA may alleviate challenges, yet coping strategies targeting financial and physical burdens are necessary.

## 1. Introduction

The incidence of cancer has been markedly increasing worldwide. In the Kingdom of Saudi Arabia (KSA), a total of 25,864 new cancer cases were reported in 2023, yet the number of newly diagnosed cancer cases is projected to rise substantially over the coming decades as a result of population growth, aging, and changes in lifestyle and risk factor profiles [1,2]. Earlier projection work estimated that the annual number of incident cancer cases among Saudi males could increase approximately six- to tenfold and among females five- to eightfold by 2030 relative to 2004 registry figures, highlighting the expected escalation in future cancer incidence [3]. In the Makkah region, 2849 cases of cancer (20%) were reported among Saudi nationals, making it the region with the second-highest number of cancer cases in the country [2].

Caregiver burden refers to the multidimensional strain experienced by individuals providing care to cancer patients, encompassing emotional distress (e.g., anxiety, depression, uncertainty about prognosis), physical fatigue from assisting with treatment side effects and daily activities, restricted social life and role conflicts, and financial demands associated with prolonged oncology care [4]. Informal caregivers may include family members—such as parents, children, and siblings—or close friends who provide unpaid, regular care to individuals with health problems or disabilities [5]. Because cancer is a physically and emotionally exhausting experience, family caregivers play a vital role in providing physical, emotional, and practical support to their loved ones throughout the illness trajectory [6,7]. In addition, caregivers may assist patients with most aspects of daily living, such as meal preparation, feeding, administering medications, bathing and dressing, toileting, and transportation [8]. As a result, caring for loved ones with cancer imposes substantial psychological, physical, social, and financial burdens [6]. Several factors have been associated with caregiver burden, including being female, low educational attainment, cohabitation with the care recipient, depression, social isolation, financial stress, prolonged caregiving hours, and the lack of autonomy in assuming the caregiving role [5].

Family caregivers of cancer patients reported experiencing some degree of burden, with burden levels varying widely from moderate to high across different studies and contexts—for example, moderate burden among Chinese family caregivers of lung cancer patients [9], higher burden among family caregivers of incurable cancer patients in Egypt [10], mild-to-moderate burden among Indian family caregivers of cancer patients receiving chemotherapy [11], and high burden among Turkish family caregivers of cancer patients [12]. However, few studies have examined caregiver burden among cancer patients in KSA, where strong cultural and familial norms place primary caregiving responsibility on family members rooted in Islamic values of familial duty and extended family networks [13]. For instance, a cross-sectional survey conducted between 2019 and 2020 among caregivers of terminally ill Saudi patients receiving palliative care at Najran University Hospital in the southern region found that 96% experienced caregiving burden, with 46% reporting mild burden, 38% moderate burden, and 11% severe burden [14]. Another survey, conducted between November 2019 and January 2021, included 244 family caregivers of patients with incurable cancer in Cairo, Egypt, and Riyadh, KSA, and found that approximately 60% of participants experienced a significant burden [10].

However, the scope of these previous studies was limited to terminally ill patients and focused only on the overall burden experienced by family members; specific types of burden were not evaluated. Therefore, the present study aimed to assess the overall caregiving burden among family caregivers of adult patients with various cancer types and stages in KSA. Additionally, it examined specific categories of caregiving burden, including psychological, social, physical, and financial aspects. Furthermore, the study explored associations between the perceived overall burden and characteristics of both caregivers and patients.

## 2. Materials and Methods

This study employed a quantitative cross-sectional design and was conducted between March and April 2024 among family caregivers of adult patients with various cancer types and stages. The study was carried out at King Abdullah Medical City (KAMC) in Makkah, KSA, a leading healthcare facility recognized for its comprehensive oncology services. The oncology department at KAMC is one of the most prominent oncology centers in KSA, offering a full spectrum of care through its medical oncology, palliative care, hematology, and radiation oncology units.

The eligibility criteria for participation included being 18 years of age or older, of either gender, and serving as a primary family caregiver—defined as the spouse, child, parent, or sibling who assumed main responsibility for providing informal (unpaid) care to a patient diagnosed with any type and stage of cancer, whether inpatient or outpatient, for at least the preceding month. In cases where multiple family members provided care, the primary caregiver was identified as the main individual most involved in day-to-day activities, symptom management, and medical coordination. Participants also needed to be willing to participate and able to speak Arabic in order to complete the questionnaire. Caregivers with psychological or mental disorders, such as cognitive impairment, were excluded from the study. Exclusion was determined through brief screening by the study researchers for obvious cognitive impairment (i.e., inability to understand study information or complete the questionnaire independently).

A convenience sampling technique was utilized by recruiting participants who were readily accessible from multiple units within the oncology center at KAMC, including the oncology clinic, the oncology and hematology units, and the chemotherapy unit. Initially, the investigators coordinated with area nurse managers and obtained approval from the head nurses of the units before recruiting. Subsequently, they approached potential and physically present family members in admission rooms in inpatient departments, chemotherapy treatment rooms in the chemotherapy department, and outpatient waiting areas in oncology and palliative care clinics to invite them to participate. Caregivers of critically ill or ICU patients were not approached for recruitment, as study personnel focused on accessible participants in the specified oncology units.

This study was performed in line with the principles of the Declaration of Helsinki. Approval was granted by the KAMC Institutional Review Board in Makkah, Saudi Arabia (Date: 28 January 2024/Number: 24-1210). Caregivers received verbal information about the study aims and procedures before enrollment and were informed of their rights, including the voluntary nature of participation and their freedom to withdraw at any time without penalty or effect on the care provided.

A link or barcode to a self-administered electronic questionnaire in Arabic was shared with those who agreed to participate, and they were asked to complete it. Participants’ responses were directly entered into an online questionnaire via SurveyMonkey, a cloud-based survey software (SurveyMonkey Inc., version 2017, San Mateo, CA, USA). The questionnaire consisted of two parts. The first part captured non-identifiable characteristics of participants, including nationality, gender, age, marital status, education level, occupation, monthly income, relationship to the patient, duration of caregiving, presence of chronic disease, absenteeism from work due to caregiving, and financial difficulties related to caregiving. It also included patient characteristics, such as insurance status, cancer stage, type of treatment, presence of pain, and mobility status. The patients’ clinical information was initially reported by family caregivers and subsequently verified using electronic medical records, as there was no direct contact with patients.

The second part collected data to assess caregiver burden using the Zarit Scale of Caregiver Burden, also known as the Zarit Burden Interview (ZBI). This scale is a well-established, reliable, and valid instrument for measuring the subjective burden experienced by caregivers, with Cronbach’s alpha values exceeding 0.97 [15,16,17]. Internal consistency was not specifically calculated for the current sample, yet the validated Arabic version has been employed in studies involving caregivers of cancer patients by several researchers [10,14,18]. The ZBI scale consists of 22 items rated on a 5-point Likert scale (0 = never, 1 = rarely, 2 = sometimes, 3 = frequently, and 4 = nearly always). The overall score, ranging from 0 to 88, is calculated by summing the values of each item. A higher score indicates a greater level of caregiver burden. Burden levels are categorized as follows: little or no burden (0–20), mild to moderate burden (21–40), moderate to severe burden (41–60), and severe burden (61–88) [19]. Although the ZBI is commonly used as a measure of overall caregiver burden, prior studies have suggested that caregiver burden can also be conceptualized as a multidimensional construct [20]. In the present study, ZBI items were grouped into four descriptive domains—psychological, social, physical, and financial burden—to provide a more detailed characterization of caregivers’ experiences [21]:

- Psychological burden is assessed using six items: stress (item 3), anger (item 5), worry about the future (item 7), strain (item 9), loss of control over one’s life (item 17), and disorientation regarding what to do (item 19). The levels are classified as low or no burden (0–8), moderate burden (9–16), and high burden (17–24).
- Social burden is assessed using three items: impact on interpersonal relationships with family or friends (item 6), interference with social activities (item 12), and discomfort in maintaining friendships (item 13). The burden levels are classified as low or no burden (0–4), moderate burden (5–8), and high burden (9–12).
- Physical burden is measured using two items: impact on physical health (item 10) and feelings of fatigue or burden (item 22). The burden levels are classified as low or no burden (0–2), moderate burden (3–5), and high burden (6–8).
- Financial burden is assessed using one item: perceived financial strain (item 15). The levels are classified as low or no burden (0–1), moderate burden (2–3), and high burden (4).

The sample size was calculated using Epi Info™ software (version 7.0, CDC, Atlanta, GA, USA), assuming a population size of 600 over a 3-month period, an expected frequency of 50%, a confidence level of 95%, a margin of error of 5%, a design effect of 1.0, and a cluster size of 1. The calculated sample size was 234. To account for potential nonresponse, an additional 10% was added, resulting in a final target sample size of 257 participants.

All data collected from the online survey platform were exported to a master Excel spreadsheet (Microsoft Office 365, version 2002, Redmond, WA, USA) for cleaning, coding, and translation. The data were then statistically analyzed using SPSS software (Statistical Package for the Social Sciences, version 26, SPSS Inc., Chicago, IL, USA). Categorical variables were summarized as frequencies and percentages, while continuous variables were presented as means and standard deviations. An independent *t*-test was used to assess differences between the means of two groups for continuous variables, and one-way ANOVA was used for comparisons among more than two groups, under the assumption of approximately normally distributed continuous variables and homogeneity of variance. Linear regression analysis was conducted to evaluate the relationship between perceived caregiver burden and one or more characteristics of caregivers and patients. For ANOVA, the *F*-statistic and corresponding *p*-value were reported, and for the independent samples *t*-test, the *t*-statistic and corresponding *p*-value were used to determine statistical significance. A *p*-value of ≤0.05 was considered statistically significant.

## 3. Results

A total of 257 family caregivers were invited to participate, of whom 212 (82%) agreed and completed the survey. Table 1 presents the demographic characteristics of the participating family caregivers and patients with cancer.

Most caregivers were Saudi nationals (88%), and half (50%) were female. Additionally, more than half of the participants (57.5%) were married, and 46% held a bachelor’s degree. More than one-third of the caregivers (34%) were the patients’ parents (either father or mother). It was also noted that more than half of the family caregivers had been providing care for more than one year and reported absenteeism from work due to caregiving responsibilities (53% and 52%, respectively). Regarding the characteristics of cancer patients, only 20% had medical insurance. More than half were diagnosed with non-metastatic cancer (stages I–III), required assistance with mobility, and were undergoing chemotherapy (57%, 51%, and 64%, respectively).

More than half of the family caregivers (55%) reported experiencing some degree of caregiving burden, of whom 35% reported mild to moderate burden (scores 21–40), 18% moderate to severe burden (41–60), and 2% severe burden (61–88). The mean overall burden score was 26.33 ± 16.86, indicating a mild to moderate burden level. Furthermore, the findings revealed that family caregivers experienced minimal levels of burden across all domains, as shown in Table 2.

Table 3 summarizes the participants’ responses to the 22 items of the ZBI. The highest mean burden scores were reported for items 7 and 21, with mean values of 2.33 ± 1.49 and 2.33 ± 1.32, respectively, whereas the lowest were for items 5 and 6, with mean values of 0.37 ± 0.83 and 0.54 ± 1.01, respectively.

Univariate statistical tests indicated a significant association between the perceived overall burden and caregivers’ financial difficulties related to caregiving, as well as between the burden and patients’ cancer stage, presence of pain, mobility status, and type of treatment (*p* < 0.05) (Table 4).

However, linear regression analysis revealed that only the patient’s mobility status and caregivers’ financial difficulties were independently associated with a significant overall burden (Table 5). Caregivers experiencing financial difficulties reported significantly higher mean burden scores than those without such difficulties (31 ± 14.8 vs. 24 ± 17.3; *p* = 0.01). Furthermore, caregivers of bedridden patients reported higher burden scores than those caring for patients with full mobility (38 ± 21.8 vs. 18 ± 12.5; *p* ≤ 0.001).

## 4. Discussion

The primary aim of this study was to evaluate the caregiving burden experienced by family caregivers of adult cancer patients in the Makkah region of KSA. The findings indicated that more than half of the caregivers reported experiencing caregiving burden. The mean ZBI score for overall burden was 26, indicating a mild to moderate burden level. This result is consistent with several studies conducted among caregivers in different countries. For instance, Unsar et al. [12], Onyeneho & Ilesanmi [22], and Zhu et al. [23] found that caregivers of cancer patients in Turkey, Nigeria, and China, respectively, experienced mild to moderate burden levels, with ZBI scores of 27, 28, and 29, respectively. In contrast, some studies have reported higher caregiving burden levels than those observed in the current study. Seo and Park [24] reported a significant burden among caregivers of advanced cancer patients in South Korea, with a mean ZBI score of 44, indicating a moderate to severe level of burden. Likewise, caregivers from Spain and Iran experienced higher burden levels, with mean ZBI scores of 52 and 55, respectively, indicating moderate to severe burden [7,25].

In comparison with studies conducted in similar cultural and geographical contexts, a survey carried out at Najran University Hospital in the southern region of KSA found that 96% of caregivers experienced some level of burden, with the majority (46%) reporting a mild burden [14]. The high burden level observed in that study may be attributed to its specific focus on terminally ill patients receiving palliative care—an area frequently associated with increased caregiver burden. Additionally, a survey conducted between 2019 and 2021 among family caregivers of patients with incurable cancer in Egypt and KSA reported a moderate burden level, with a mean ZBI score of 23 [10]. This is comparable to the present findings and reinforces the consistency of mild to moderate burden levels in similar cultural and geographic settings. It is important to note that both previous studies concentrated on the challenges faced by caregivers of patients in a metastatic stage of cancer. In contrast, the current study examined the caregiving burden across all stages of cancer, and findings revealed that 57% of the patients were in non-metastatic stages and 64% were receiving chemotherapy.

Caregivers in the present study reported low levels of all burden subtypes. This contrasts with other studies that have identified significant psychosocial and financial burdens. For instance, García-Torres et al. [26] reported moderate levels of anxiety and depression among caregivers, while Grunfeld et al. [27] found high levels of these symptoms among caregivers of breast cancer patients. In 2018, a survey revealed that caregivers of patients with oral cancer experienced substantial social burdens, particularly regarding loss of privacy and discomfort in maintaining friendships [28]. Financial burden was also found to be significant in several studies, which reported major financial strain [18,27]. These variations may be explained by differing caregiver and patient characteristics, disease stages, or health system factors. The comprehensive healthcare system in KSA provides free medical services, thereby alleviating financial stress for patients and their families. In contrast, countries with less comprehensive healthcare coverage may place a greater financial burden on caregivers, which can, in turn, lead to increased psychological and physical stress.

Furthermore, the present study revealed that the ZBI item most strongly associated with fear regarding the patient’s future had the highest mean score. Both Lins et al. [29] and Amirthraj et al. [28], who studied Brazilian and Indian primary caregivers of cancer patients, respectively, reported significant concerns about the future and the adequacy of the care they provided. Conversely, the ZBI items related to feelings of anger when in the presence of the patient and to the patient’s negative impact on other relationships had the lowest mean scores. This suggests that although caregivers may experience psychological burden, they may not encounter high levels of anger or disruptions in other personal relationships. Such findings may reflect the influence of strong cultural values and social support from both family and professional institutions. Saudi culture and Islamic beliefs place considerable emphasis on maintaining close family ties and offering mutual support, which can help mitigate the emotional stress associated with caregiving. For instance, Saudi caregivers of parents undergoing hemodialysis perceived caregiving as a religious duty and believed that their sacrifices would be divinely rewarded with greater happiness and blessings [30].

Financial difficulties were significantly associated with caregiver burden in the present study, consistent with prior research. For instance, a survey among parents of children with cancer in Jordan found that financial hardship was associated with higher stress levels, highlighting the substantial impact of economic challenges on caregiving [18]. This burden may be explained by both direct and indirect costs, including transportation, special diets, accommodation, reduced work hours or job loss, and out-of-pocket healthcare expenses such as advanced treatments or specialized private care [27].

Regression analysis in the present study also demonstrated a significant association between patients’ mobility status and caregiver burden. Similarly, Alsirafy et al. [10] reported increased burden associated with assisting patients in performing basic activities of daily living, such as eating, bathing, and mobility. Another study identified a strong association between declining patient functional status—measured by the Eastern Cooperative Oncology Group performance scale—and increased caregiver burden among Turkish caregivers [12]. Caregiving tasks such as repositioning patients, managing personal hygiene, and addressing incontinence in bedridden individuals can further exacerbate physical strain, potentially leading to chronic pain and musculoskeletal injuries, while also intensifying emotional stress, anxiety, and depression [31].

The burden observed in this study highlights the need for coping mechanisms to help manage the physical, emotional, and psychological stress associated with caregiving. Prior evidence suggests that social support from friends, family, and support groups, along with practical strategies such as scheduling, task delegation, and problem-solving, may help reduce caregiver burden [32]. Additionally, religiosity and spiritual practices may provide emotional resilience [33], while psychological interventions such as psychoeducational programs and cognitive behavioral therapy have been shown to improve mental health outcomes [34].

Some limitations were present in this study. The study was confined to a single geographic region in KSA, which may limit the generalizability of the findings to other regions in the country. Moreover, the use of convenience sampling from specific oncology units at a single center may limit generalizability. Caregivers of critically ill or ICU patients and those not physically present at the hospital during recruitment periods were not included, potentially underrepresenting caregivers facing the highest burden levels. Although the calculated sample size indicated a target of 257 participants, only 212 family caregivers completed the survey. This shortfall may have reduced the statistical power to detect smaller associations and should be considered when interpreting the findings. Also, the self-reported nature of the questionnaire may have introduced response bias, as participants could underreport or overreport their experiences based on personal perceptions, social desirability, or acquiescence bias. In addition, caregivers’ relationship with the healthcare system and the cultural framing of caregiving as a familial duty rather than a burden may have contributed to underreporting the true extent of caregiver burden. Lastly, the cross-sectional design precludes the examination of changes in caregiver burden over time, thereby limiting understanding of how caregiver stress evolves with disease progression.

For future research, larger and more diverse samples should be involved to enhance the generalizability of results across different regions and populations in KSA. Longitudinal studies are warranted to assess how caregiver burden evolves and to examine the long-term effects on caregivers’ physical and mental health. In addition, the use of qualitative research methods—such as in-depth interviews and focus groups—can provide richer insights into caregivers lived experiences and the specific challenges they face.

These findings have important clinical and policy implications for improving support for family caregivers of cancer patients. Clinically, the results emphasize the need for integrated support services that include counseling, mental health care, and caregiver education. Routine screening with tools such as the ZBI is essential to identify caregivers at risk and to guide timely intervention. Expanding access to telehealth services may also provide caregivers with more flexible and accessible support and information. From a health policy perspective, the findings support the development of structured respite care services to temporarily relieve primary caregivers, reduce burnout, and preserve care quality. Financial assistance programs are also suggested to help families manage out-of-pocket transportation and housing costs, particularly for those traveling long distances for treatment. Moreover, an online information and resources hub, supported by case management services, could improve access to available support, while workplace flexibility, including remote work options, may help employed caregivers better balance caregiving and occupational responsibilities.

## 5. Conclusions

This study examined the caregiving burden among family caregivers of adult cancer patients in Makkah, KSA, revealing that over half experienced mild to moderate burden levels. Unlike previous research, the reported burden was relatively low, likely due to strong cultural and familial support systems. Patient immobility and financial difficulties were the key factors linked to increased burden. This study emphasizes the need for tailored coping strategies and recommends future research with more diverse samples and qualitative approaches to enhance caregiver support and well-being.

## Figures and Tables

**Table 1:** Characteristics of family caregivers and cancer patients (*n* = 212).

Type of Variables	Variable Categories	*n* (%)
Caregivers’ Characteristics		
Nationality	Saudi	187 (88)
	Non-Saudi	25 (12)
Gender	Male	106 (50)
	Female	106 (50)
Age group	18–29	50 (24)
	30–39	76 (36)
	40–49	45 (21)
	Above 50	41 (19)
Marital status	Single	75 (35)
	Married	122 (58)
	Divorced	12 (6)
	Widowed	3 (1.0)
Educational level	No Education	5 (2.0)
	Less than high school	18 (9.0)
	High School	54 (26)
	Diploma	20 (9.0)
	Bachelors	97 (46)
	Postgraduate	18 (9.0)
Occupation	Student	22 (10)
	Unemployed	53 (25)
	Government Sector	80 (38)
	Private Sector	34 (16)
	Self-employed	7 (3.0)
	Retired	16 (8.0)
Monthly Income	Less Than 5000 SAR	74 (35)
	5000–10,000 SAR	71 (34)
	10,000–15,000 SAR	42 (20)
	Above 15,000 SAR	25 (12)
Relation to the patient	Parent (father or mother)	73 (34)
	Child (son or daughter)	60 (28)
	Sibling (brother or sister)	42 (20)
	Spouse (husband or wife)	37 (18)
Duration of caregiving	≤1 year	100 (47)
	˃1 year	112 (53)
Having a chronic disease	Yes	68 (32)
	No	144 (68)
Absenteeism from work due to caregiving	Yes	111 (52)
	No	101 (48)
Having financial difficulties due to caregiving	Yes	63 (30)
	No	149 (70)
**Patients’ characteristics**		
Holding medical insurance	Yes	43 (20)
	No	169 (80)
Stage of Cancer	Non-metastatic (stages I–III)	121 (57)
	Metastatic (stage IV)	67 (32)
	End-of-life stage	24 (11)
Type of cancer treatment	Chemotherapy	135 (64)
	Surgery	10 (5.0)
	Palliative care	24 (11)
	Other treatments (e.g., immunotherapy, radiation therapy, hyperthermia)	34 (16)
	No treatment	9 (4.0)
Patient having pain	Yes	166 (78)
	No	46 (22)
Patient mobility status	Bedridden	28 (13)
	With assist	107 (51)
	Full movement	77 (36)

SAR: Saudi Riyal.

**Table 2:** Mean scores for types of burden among family caregivers of cancer patients (*n* = 212).

Type of Burden	Min-Max	Mean ± SD	Level of Burden
Psychological burden	0–23	7.34 ± 5.41	No to low burden
Social burden	0–12	2.27 ± 2.93	No to low burden
Physical burden	0–8	1.96 ± 2.22	No to low burden
Financial burden	0–4	1.22 ± 1.41	No to low burden
**Overall burden**	0–88	26.33 ± 16.86	Mild to moderate burden

**Table 3:** Frequencies and mean scores for caregiving burden items among family caregivers of cancer patients (*n* = 212).

Items	Never *n* (%)	Rarely *n* (%)	Sometimes *n* (%)	Frequently *n* (%)	Nearly Always *n* (%)	Mean ± SD
Do you feel the patient ask for more help than he/she needs?	78 (36.8)	45 (21.2)	56 (26.4)	20 (9.4)	13 (6.1)	1.27 ± 1.22
2. Do you feel you don’t have enough time for yourself?	92 (43.4)	34 (16.0)	54 (25.5)	21 (9.9)	11 (5.2)	1.17 ± 1.23
3. Do you feel stressed of fulfilling different responsibilities?	78 (36.8)	43 (20.3)	44 (20.8)	30 (14.2)	17 (8.0)	1.36 ± 1.31
4. Do you feel embarrassed of patient behavior?	143 (67.5)	30 (14.2)	26 (12.3)	10 (4.7)	3 (1.4)	0.58 ± 0.97
5. Do you feel angry when you are around your patient?	168 (79.2)	20 (9.4)	15 (7.1)	7 (3.3)	2 (0.9)	0.37 ± 0.83
6. Do you feel negative effect on other relationships?	155 (73.1)	17 (8.0)	28 (13.2)	6 (2.8)	6 (2.8)	0.54 ± 1.01
7. Do you feel afraid of patient’s future?	46 (21.7)	12 (5.7)	41 (19.3)	52 (24.5)	61 (28.8)	2.33 ± 1.49
8. Do you feel patient is too dependent?	52 (24.5)	28 (13.2)	56 (26.4)	35 (16.5)	41 (19.3)	1.93 ± 1.43
9. Do you feel strained around patient?	147 (69.3)	27 (12.7)	17 (8.0)	14 (6.6)	7 (3.3)	0.62 ± 1.09
10. Do you feel your health affected by caregiving?	116 (54.7)	26 (12.3)	41 (19.3)	21 (9.9)	8 (3.8)	0.96 ± 1.21
11. Do you feel having inadequate privacy?	143 (67.5)	26 (12.3)	28 (13.2)	12 (5.7)	3 (1.4)	0.61 ± 1.00
12. Do you feel suffering in social life?	116 (54.7)	23 (10.8)	40 (18.9)	22 (10.4)	11 (5.2)	1.00 ± 1.27
13. Do you feel uncomfortable having friends?	137 (64.6)	23 (10.8)	32 (15.1)	12 (5.7)	8 (3.8)	0.73 ± 1.13
14. Do you feel patient expected you to be the only caregiver?	66 (31.1)	26 (12.3)	48 (22.6)	28 (13.2)	44 (20.8)	1.80 ± 1.51
15. Do you feel financially stressed?	105 (49.5)	23 (10.8)	36 (17.0)	28 (13.2)	20 (9.4)	1.22 ± 1.41
16. Do you feel unable to take care of the patient much?	141 (66.5)	35 (16.5)	20 (9.4)	6 (2.8)	10 (4.7)	0.63 ± 1.07
17. Do you feel sense of losing control over life?	96 (45.3)	31 (14.6)	48 (22.6)	23 (10.8)	14 (6.6)	1.19 ± 1.29
18. Do you feel wish to leave caring of the patient?	126 (59.4)	24 (11.3)	45 (21.2)	8 (3.8)	9 (4.2)	0.82 ± 1.14
19. Do you feel uncertain of what to do?	81 (38.2)	32 (15.1)	46 (21.7)	23 (10.8)	30 (14.2)	1.48 ± 1.44
20. Do you feel should be doing more for the patient?	49 (23.1)	14 (6.6)	42 (19.8)	42 (19.8)	65 (30.7)	2.28 ± 1.53
21. Do you feel could do better for the patient?	28 (13.2)	25 (11.8)	63 (29.7)	42 (19.8)	54 (25.5)	2.33 ± 1.32
22. Do you feel burdened of caring?	109 (51.4)	31 (14.6)	30 (14.2)	26 (12.3)	16 (7.5)	1.10 ± 1.35

**Table 4:** Mean differences in family caregivers’ burden in relation to caregivers and patient characteristics (*n* = 212).

Type of Variables	Variable Categories	Total Burden		
		Mean ± SD	*t* or *f*	*p*-Value
**Caregivers’ Characteristics**				
Nationality	Saudi	26.47 ± 16.42	0.33	0.74
	Non-Saudi	25.28 ± 20.18		
Gender	Male	25.49 ± 16.78	0.72	0.46
	Female	27.17 ± 16.98		
Age group	18–29	24.98 ± 16.76	0.86	0.46
	30–39	25.07 ± 16.54		
	40–49	26.71 ± 16.60		
	Above 50	29.90 ± 17.90		
Marital Status	Single	25.94 ± 15.72	0.16	0.92
	Married	26.22 ± 17.44		
	Divorced	29.58 ± 19.36		
	Widowed	27.33 ± 17.89		
Educational level	No Education	37.60 ± 13.04	0.69	0.63
	Less than high school	28.05 ± 17.56		
	High School	25.72 ± 16.88		
	Diploma	27.85 ± 17.06		
	Bachelors	25.07 ± 17.01		
	Postgraduate	28.44 ± 16.57		
Occupation	Student	20.18 ± 11.57	1.41	0.22
	Unemployed	28.0 ± 16.98		
	Government Sector	25.45 ± 16.69		
	Private Sector	30.32 ± 18.59		
	Self-employed	19.28 ± 16.70		
	Retired	28.31 ± 18.42		
Monthly Income	Less Than 5000 SAR	26.32 ± 17.94	0.24	0.86
	5000–10,000 SAR	27.45 ± 17.98		
	10,000–15,000 SAR	24.66 ± 13.64		
	Above 15,000 SAR	26.0 ± 15.82		
Relation to the patient	Parent (father or mother)	23.04 ± 15.97	2.0	0.11
	Child (son or daughter)	27.15 ± 15.36		
	Sibling (brother or sister)	30.85 ± 19.60		
	Spouse (husband or wife)	26.37 ± 16.88		
Duration of caregiving	≤1 year	25.29 ± 16.25	0.85	0.39
	˃1 year	27.26 ± 17.41		
Having a chronic disease	Yes	29.39 ± 17.57	1.82	0.06
	No	24.88 ± 16.38		
Absenteeism from work due to caregiving	Yes	28.03 ± 17.84	1.54	0.12
	No	24.46 ± 15.59		
Having financial difficulties due to caregiving	Yes	30.96 ± 14.83	2.63	0.009 *
	No	24.37 ± 17.32		
**Patients’ characteristics**				
Holding medical insurance	Yes	24.74 ± 15.94	0.69	0.49
	No	26.73 ± 17.11		
Stage of Cancer	Non-metastatic (stages I–III)	22.10 ± 14.44	9.62	<0.001 *
	Metastatic (stage IV)	31.64 ± 17.40		
	End-of-life stage	32.83 ± 20.78		
Type of cancer treatment	Chemotherapy	24.01 ± 14.96	5.44	<0.001 *
	Surgery	20.00 ± 11.83		
	Palliative care	35.66 ± 19.45		
	Other treatments (e.g., immunotherapy, radiation therapy, hyperthermia)	33.35 ± 19.34		
	No treatment	16.77 ± 15.69		
Patient having pain	Yes	28.21 ± 16.71	3.14	0.002 *
	No	19.56 ± 15.77		
Patient mobility status	Bedridden	38.35 ± 21.77	19.9	<0.001 *
	With assist	28.91 ± 15.56		
	Full movement	18.37 ± 12.50		

SAR: Saudi Riyal; t: Independent Samples Test; f: One-way ANOVA test; * Significant at *p* ≤ 0.05.

**Table 5:** Relationship between family caregivers’ and patients’ characteristics and overall burden (*n* = 212).

Variables	*p*-Value	95% CI	
		Lower	Upper
**Caregivers’ Characteristics**			
Age	0.21	−1.00	4.37
Gender	0.45	−2.95	6.53
Nationality	0.60	−9.41	5.48
Marital status	0.23	−6.64	1.63
Educational level	0.76	−2.17	1.59
Occupation	0.60	−1.45	2.49
Monthly income	0.87	−2.69	2.29
Relation to the patient	0.06	−0.09	3.93
Duration of caregiving	0.72	−5.18	3.59
Having a chronic disease	0.40	−6.74	2.71
Absenteeism from work due to caregiving	0.21	−7.26	1.60
Having financial difficulties due to caregiving	0.01 *	−11.77	−1.53
**Patients’ characteristics**			
Holding medical insurance	0.50	−3.44	7.01
Stage of Cancer	0.26	−1.51	5.52
Type of cancer treatment	0.20	−0.43	2.00
Patient having pain	0.36	−8.08	2.96
Patient mobility status	0.001 *	−11.95	−4.41

* Significant at *p* ≤ 0.05; CI = Confidence Interval.

## Data Availability

The data presented in this study are available on request from the corresponding author. The data are not publicly available due to privacy and ethical restrictions, as they contain information collected from human participants and are subject to institutional ethical approval conditions.
